# 

**Published:** 1894-09-29

**Authors:** 


**Notices of Births, Deaths, and Marriages** are inserted in this column at a charge of 2s. 6d. for an announcement not exceeding four lines.

**Notice to Advertisers and Subscribers.**

All Advertisements, Orders for Copies of The Hospital, and Business Communications should be addressed to **The Publisher, NOT TO THE EDITOR,** The Hospital, 428, Strand, W.C.

The Cost of Subscription, post free, is as follows:—Per week, 2½d.; per quarter, 2s. 8d.; per half-year, 5s. 3d.; per annum, 10s. 6d. Foreign:—Per Annum, 12s. 8d.; payable, in all cases, in advance.

### NOTICE TO CORRESPONDENTS.

All MS., Letters, Books for Review, and other matters intended for the Editor should be addressed The Editor, The Lodge, Porchester Square, London, W.

The Editor cannot undertake to return rejected MS., even when accompanied by stamped directed envelope.

**"Burdett's Hospital Annual," 1894.**

Fifth year of publication. 900 pp. Boards, gilt lettering. A most complete guide to British, Colonial, Indian, and American Hospitals, Dispensaries, Nursing and Convalescent Institutions, and Asylums. Price 5s. Published by The Scientific Press (Limited), 428, Strand, W.C.

**NOTICE.—The Index for Vol. XV. (ending March 31st, 1894) is on sale at the Office of this Journal, price 2½d., post free.**

### SCRAPS AND GLEANINGS.

Hospital Saturday this year at Sidcup proved the same financial success as last year.

The Anatomical Congress will hold its next meeting at Basle (Switzerland) on April 17th, 1895.

On behalf of local hospitals £54 was collected at Tunbridge Wells on the occasion of Hospital Saturday.

The Duke and Duchess of Portland have opened a cottage hospital for the benefit of their tenants and servants at Berridelen.

We learn that the Apollinaris Mineral water have received the highest award at the Antwerp Exhibition—the Diplome d'Honneur.

In order to enlarge the Cochin Hospital in Paris, three houses contiguous to the hospital in the Faubourg St. Jacques have been acquired for the purpose of extension.

A new Cottage Hospital has been opened at Dumfermline. The committee start with a guarantee of £200 towards the maintenance fund, which is projected to be about £600 a year.

As a result of the garden party organised by the Employés Committee at Manningham Park in August last, a sum of £74 2s. 7d. has been handed over to the Bradford Joint Hospital Fund.

A great effort was put forth this year in Exeter to raise £1,000 for the Hospital Saturday Fund. The ladies showed an energetic interest in the cheme, and £158 was contributed through their endeavours.

A meeting in aid of the National Society for the Prevention of Cruelty to Children was held by permission of Miss Pater, at Bowes Park, N. This estimable society is unhappily over £5,000 in debt, and in great need of funds.

Hospital Sunday at Penryn proved especially successful this year, partly owing, no doubt, to a very fine day. The amount collected at the service at the Wesleyan Church, which was largely attended, amounted to £5 2s. 3d.

An anonymous donor has offered £1,000 for two wards in the Blackpool Hospital, one for males and one for females. These to be built, furnished, and fully equipped without delay, each to be capable of accommodating ten patients.

The Friendly Societies' Hospital Parade held its annual meeting at Barnet. The collection which was made was instituted to benefit the Jubilee Cottage Hospital, erected at the Queen's special command for Barnet and the neighbourhood.

Three streets in Paris have lately been named after the eminent doctors—Charcot, Trelat, and Trousseau. The Municipal Council of the French capital have for some years adopted this excellent plan of memorialising great scientists.

The memorial stone of the first Consumption Home for Scotland has recently been laid, the site selected being at the Bridge of Weir. The estimated cost of the first hospital building is £7,000, and the accommodation provides from 35 to 50 beds.

The Convalescent Home at L'Ancrerse Common, Guernsey, is greatly in want of funds, and the committee report that unless an increase of subscriptions can be obtained the committee are afraid they will have to close the doors of this most useful institution.

The Sheffield Infirmary adds yearly to its record of good. Last year 900 more cases were treated in the institution than in 1893. An appeal in aid of the funds is to be made. The income is seriously below the needs of the infirmary by no less a sum than £2,987.

During the present military manœuvres in Germany, in which the German Emperor has taken so active a part, it is reported that the "disabled" soldiers have had rather a bad time. Hospital and ambulance provision has, of course, been made, but not on a very large scale.

The ambulance vehicles and appliances which comprise part of the exhibit of the War Office at the Lyons Exhibition are of an interesting description. Six carriages of different kinds comprise the collection, containing all the conveniences possible for tending the wounded in war.

A large gathering of ladies and gentlemen of the surrounding country were invited recently to inspect the buildings and arrangements of the Newhill Home for Convalescents, Aberdeenshire. During the 18 years that the home has been in existence 2,000 invalids have benefited by it.

The Committee of the Wolverhampton and Staffordshire General Hospital have decided to make a most earnest appeal to the generosity of the people of Wolverhampton and the surrounding neighbourhood for funds to carry out the necessary scheme of supplying a mortuary and other accessory buildings to the hospital.

At a recent monthly meeting of the Committee of Management of the Swansea Hospital, it was announced that Sir John Llewelyn, Bart., had decided to apply the income of a certain fund, amounting to some £1,200, towards the benefit of the hospital funds. A cordial vote of gratitude and thanks was passed.

The increased number of patients at the Bristol General Hospital has involved an additional outlay of funds, more than £375 having been spent during the last half-year than was the case in the corresponding one. Attention has been called to the necessity for an extension to the hospital.

A Medical Congress will be held in Calcutta, commencing on September 24th. It will be divided into six sections—Medicine and Pathology; Surgery and Ophthalmology; Obstetrics and Diseases of Women and Children; Public Medicine; Legal Medicine and Insanity; Pharmacy and Special Drugs.

Through the kindness of the Countess of Mayo, a furnished house adjoining Palmerston desmesne was placed this summer at the disposal of the patients of the Orthopædic Hospital of Dublin. Many alterations and improvements were effected in the institution during the temporary absence of the patients.

Cycling for women is occupying great attention at present. A French medical journal has formulated certain inquiries on the subject, upon which well-known doctors of several countries are asked to give their opinion. The general opinion seems to be that the exercise is good, but when carried to an excess it is bad.

The Sanitary Institute holds its Autumn Congress this year at Liverpool, and opens on September 24th. It will begin by a reception of the members of the Congress of the Lord Mayor of Liverpool, and will end on September 28th by an address to the working classes by Sir James Crichton-Browne. Saturday, the 29th, will be devoted to excursions.

### Books Received.

Young J. Pentland.
"Prescribing and Treatment in the Diseases of Infants and Children." By Philip E. Muskett.

Cassell and Co.
"Diet and Cookery for Common Ailments." By a Fellow of the Royal College of Physicians and Phyllis Browne.

J. and A. Churchill.

"Lunacy Law for Medical Men." By Charles Mercier, M.B.

William Rider and Son.

"A Healthy Home." By Francis Vacher.

Simpkin, Marshall, Hamilton, Kent, and Co.
"Physical Culture for Men, Women, and Children." By Arthur E. Tanner.

Scientific Press.
"Science Progress." Vol. I. March—August, 1894.
"Medical History from the Earliest Times." By E. T. Withington, M.A., M.B.Oxon.
"Nursing: Its Principles and Practice." By Isabel Adams Hampton.

British and Colonial Druggist.
"Minor Ailments." By a Medical Practitioner.
"Practical Dispensing for Students." By C. J. S. Thompson.

Thomas Brear and Co., Bradford.
"Ethics in Medicine." By a West Riding Practitioner.

Drake, Driver, and Leaver.
"Asylum Construction and Arrangement." By George R. Bibby, F.R.I.B.A.

**Periodicals and Pamphlets.**—Review of Reviews, Chicago Clinical Review, Therapeutic Gazette, The Sun, The Therapist, English Illustrated Magazine, Sunday at Home, Leisure Hour, Girl's Own Paper, Boy's Own Paper, London, The Vegetarian, Strand Magazine, Tit-Bits, Illustrated Penny Tales, Picture Magazine, The Million, and Pal's Puzzles.

### The Student of Medicine.

### APPOINTMENTS.

J. Acheson-Wilkin, L.R.C.P. and S.Edin., L.F.P.S.Glasg., L.M., appointed Assistant Surgeon, East Dispensary, Liverpool. T. A. Anderson, M.D.Edin., appointed Medical Officer of the Isolation Hospital of the Epsom Local Board. W. L. Christie, M.D., M.R.C.S., appointed House Surgeon to the Bristol Hospital for Sick Children and Women. F. W. Clark, M.B.Durh., L.R.C.P.Lond., reappointed Medical Officer of Health for the Borough and Port of Lowestoft and Medical Superintendent to the Lowestoft Sanatorium. H. Collier, M.D.Brux., M.R.C.S. Eng., L.R.C.P.Edin., appointed Medical Officer for the South District of the Great Yarmouth Union. Dr. F. Ellison, appointed Resident Assistant Medical Officer to the Mayo Lunatic Asylum. T. W. Hicks, M.B.Lond., L.R.C.P., M.R.C.S., appointed Medical Officer of the Highgate Division of the Great Northern Railway and Medical Officer to the Sixth District of the Barnet Union. W. Hoffmeister, M.D.Heidelb., reappointed Medical Officer of Health to the Cowes Port Sanitary Authority. A. A. Martin, M.R.C.S., L.R.C.P., late House Surgeon to St. Mary's Hospital, W., appointed Third Assistant Medical Officer to the Hants County Asylum, Fareham. Ahmed Mirza, M.B., C.M., B.Sc.Edin., appointed Medical Officer of Health for the City of Hyderabad, Deccan, India. R. H. Parry, L.R.C.P., L.R.C.S. Edin., appointed Honorary Surgeon to the Royal Hospital for Sick Children, Glasgow. T. Pettey, M.B., C.M.Edin., M.R.C.S. Eng., L.R.C.P.Lond., appointed Assistant House Surgeon to the General Infirmary at Gloucester and the Gloucestershire Eye Institution. J. Robertson, M.D.Edin., appointed Medical Officer of Health for the St. Helen's Urban Sanitary District. R. H. Shaw, M.R.C.S., L.R.C.P.Lond., appointed Medical Officer of Health for the Liversedge Urban Sanitary District. D. E. Thomas, M.R.C.S.Eng., L.S.A., appointed Medical Officer for the Christchurch and Nash Districts of the Newport Union. F. K. Tweedie, M.B.R.U.I., B.Ch., reappointed Medical Officer of Health to the Hucknall Huthwaite Local Board. C. H. Usher, M.B.Cantab., F.R.C.S.Edin., appointed Ophthalmic Surgeon to the Aberdeen Royal Infirmary. J. D. Windle, L.R.C.P.I., L.R.C.S.Edin., reappointed Medical Officer of Health to the Southall and Norwood Urban Sanitary Districts.

### VACANCIES.

The following vacancies are announced this week, the amount of salary in each case being given after the name of the institution:

**Resident Medical Officers.**

Bethlem Hospital.—Two Resident Clinical Assistants. Apartments, rations, and attendance provided. Applications, endorsed "Clinical Assistantship," to the Treasurer by October 1st.

Cambridge University.—John Lucas Walker Studentship. Applications to Professor Roy, New Museums, Cambridge, by September 30th.

THE STUDENT OF MEDICINE—*continued.*

Cancer Hospital (Free), Brompton, S.W.—Two House Surgeons. Salary at the rate of £60 and £50 per annum respectively, with board and residence. Applications to the Secretary by October 5th.

Cumberland Infirmary, Carlisle.—Assistant House Surgeon. Salary £40 per annum, with board, lodging, and washing. Appointment for one year. Applications to the Secretary by October 9th.

East London Hospital for Children, Glamis Road, Shadwell, E.—House Physician. Board, lodging, &c., provided, but there is no salary attached to the post. Applications to the Secretary by October 6th.

General Infirmary, Birmingham.—Resident Surgical Officer and House Physician. Salary £130 and £70 per annum respectively, with residence, board, and washing. Appointments for one year, but candidates will be eligible for re-election for two subsequent years. Also two Assistant House Surgeons, appointment for six months, but eligible for re-election for another six months. No salary. Residence, board, and washing provided. Applications to Howard J. Collins, House Governor, by October 6th.

Leith Hospital.—House Physician, House Surgeon, and Surgeon for the Outdoor Department. Appointments for six months. Salaries at the rate of £50 a year each, with board in the hospital. Applications to the Secretary, Mr. George V. Mann, 33, Bernard Street, Leith, by October 1st.

Manchester Clinical Hospital for Women and Children, Park Place, Cheetham Hill Road.—House Surgeon. Salary £80 per annum, with apartments and board. Applications to Hubert Teague, Secretary, 38, Barton Arcade, Manchester, by October 3rd.

Metropolitan Hospital, Kingsland Road, N.E.—House Physician, House Surgeon, and Assistant House-Surgeon. Appointments from November 1st, 1894, to March 31st, 1895. Salary at the rate of £60 a year for the two former, and at the rate of £30 a year for the latter. Applications to Charles H. Byers, Secretary, by October 1st.

St. Marylebone General Dispensary, 77, Welbeck Street, Cavendish Square, W.—Resident Medical Officer, doubly qualified. Salary 100 guineas per annum, with an annual increase of 10 guineas to a maximum of 120 guineas, with furnished apartments, attendance, coal, and gas. Applications to the Secretary by October 1st.

Seamen's Hospital Society, "Dreadnought," Greenwich, S.E.—House Physician and House Surgeon to the Dreadnought Hospital. Salary £75 and £50 per annum respectively, with board and residence. Also Junior House Surgeon for Branch Hospital, Royal Albert Dock, E. Salary £50 per annum, with board and residence. Applications to P. Michelli, Secretary, by October 3rd.

The Coppice Asylum, Nottingham.—Assistant Medical Officer; unmarried, and not exceeding 28 years of age. Salary £120 per annum, with apartments, board, attendance, and washing. Applications to Dr. Tate, at the Asylum.

Victoria Infirmary of Glasgow.—Superintendent and Resident Medical Officer. Salary £300 per annum, with board and lodging in the infirmary. Applications to Francis Bisset, Secretary, by October 3rd.

### Non-Resident Medical Officers.

Clutton Union.—Medical Officer of Health to the Sanitary Authority. Salary £100 per annum. Applications to Edward H. Perrin, Solicitor, Clerk to the Rural Sanitary Authority, Temple Cloud, near Bristol, by October 3rd.

Dental Hospital of London, Leicester Square.—Assistant Dental Surgeon. Must be Licentiate of Dental Surgery of one of the licensing bodies recognised by the General Medical Council. Applications and testimonials to J. Francis Pink, Secretary, by October 8th.

The General Infirmary at Gloucester and Gloucestershire Eye Institution.—Assistant Surgeon; must be F.R.C.S. or M.R.C.S.Eng., or F.R.C.S. or L.R.C.S. of Ireland or of Edinburgh, or graduate of University recognised by the General Medical Council. Applications and testimonials to Henry T. Pike, Secretary, by October 10th.

### Medical Appointments.

SEAMEN'S HOSPITAL SOCIETY.
DREADNOUGHT, GREENWICH, S.E.

The following MEDICAL OFFICERS are REQUIRED for the service of the Society. Candidates must be doubly qualified and registered:—

HOUSE PHYSICIAN for Dreadnought Hospital, Greenwich. Salary £75 per annum, with Board and Residence.

HOUSE SURGEON for Dreadnought Hospital, Greenwich. Salary £50 per annum, with Board and Residence.

JUNIOR HOUSE SURGEON for Branch Hospital, Royal Albert Dock, E. Salary £50 per annum, with Board and Residence.

